# Supplementary figures and images for: Early indicators of intensive care unit bed requirement during the COVID-19 epidemic: A retrospective study in Ile-de-France region, France
Source: PLoS One. 2020 Nov 18;15(11):e0241406. doi: 10.1371/journal.pone.0241406 (PMC7673527; doi:10.1371/journal.pone.0241406)

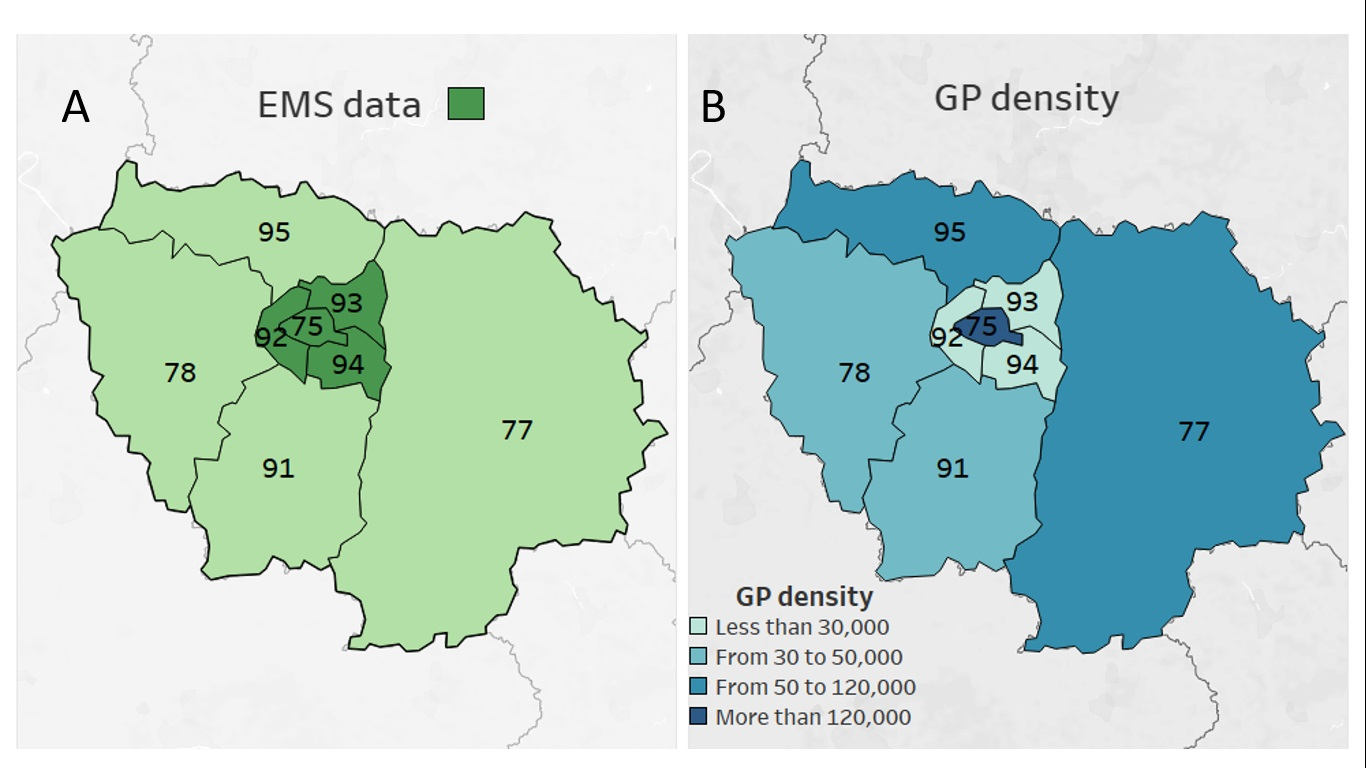

Supplement: S1 Fig — This French region (12·1 million inhabitants) comprises eight administrative sub-identities, indicated by their number on the map, the town of Paris being 75. In the present study, the numbers of emergency departments (ED) visits, positive reverse transcriptase polymerase chain reaction (RT-PCR) tests, hospital admissions, intensive care unit (ICU) patients, and new ICU patients were obtained from the whole region. A regionalized organization was installed enabling to rapidly find an ICU bed for a given patient wherever the patient was initially admitted. Panel A: Data from emergency medical system (EMS), including emergency calls and dispatch of ambulances were obtained from the Paris city (75) and its inner suburbs which comprise four administrative sub-entities (75, 92, 93, 94) and their respective EMS (6·71 million inhabitants). Panel B: Data from general practitioner (GP, SOS Médecins network) were obtained from the Ile-de-France region but the density of activity of this GP network (expressed as number of annual visits per million inhabitants).is heterogeneous within the Ile-de-France region. (TIF) [file pone.0241406.s001.tif]

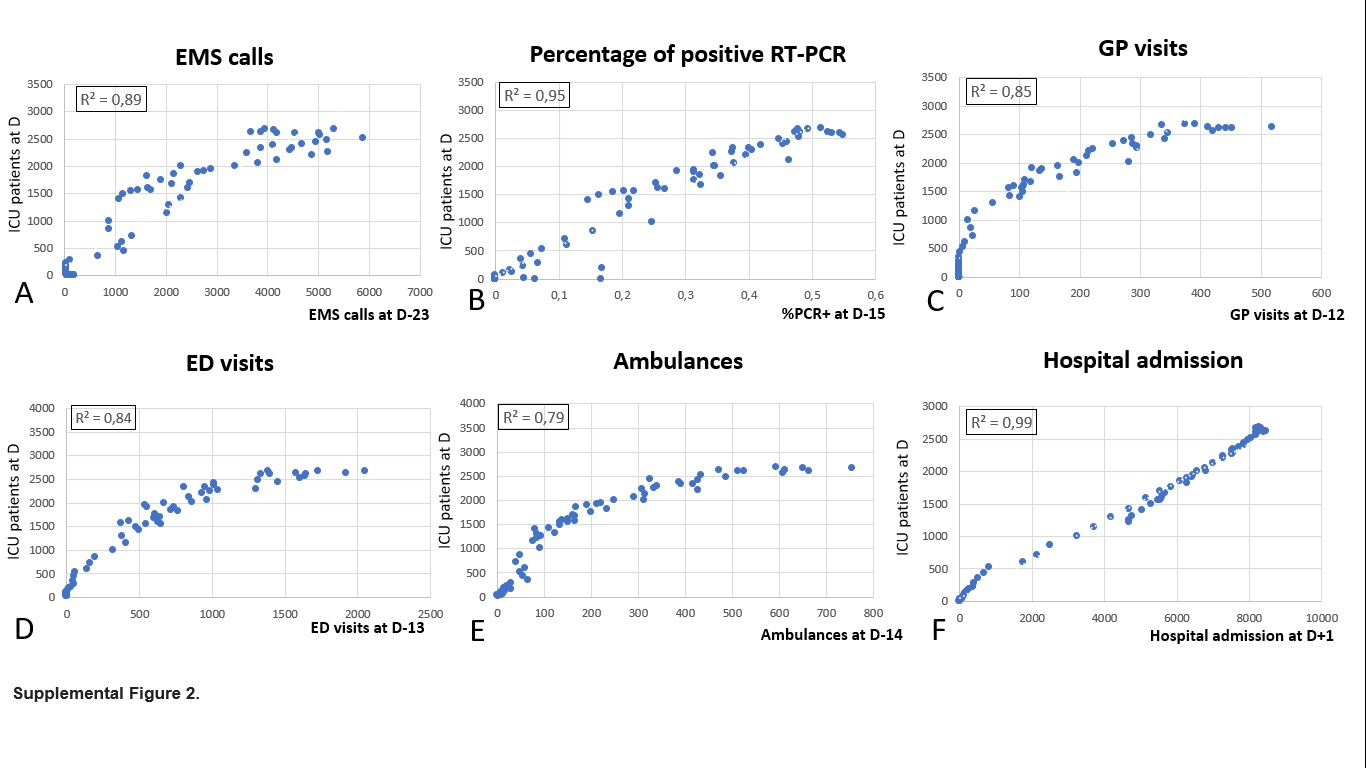

Supplement: S2 Fig — Correlation curves of the six tested indicators compared to the number of intensive care unit (ICU) patients during the study period. EMS: emergency calls; GP: general practitioner; ED: emergency department. D: delay (in days) between the two variables. R2: Pearson coefficient of correlation. (TIF) [file pone.0241406.s002.tif]

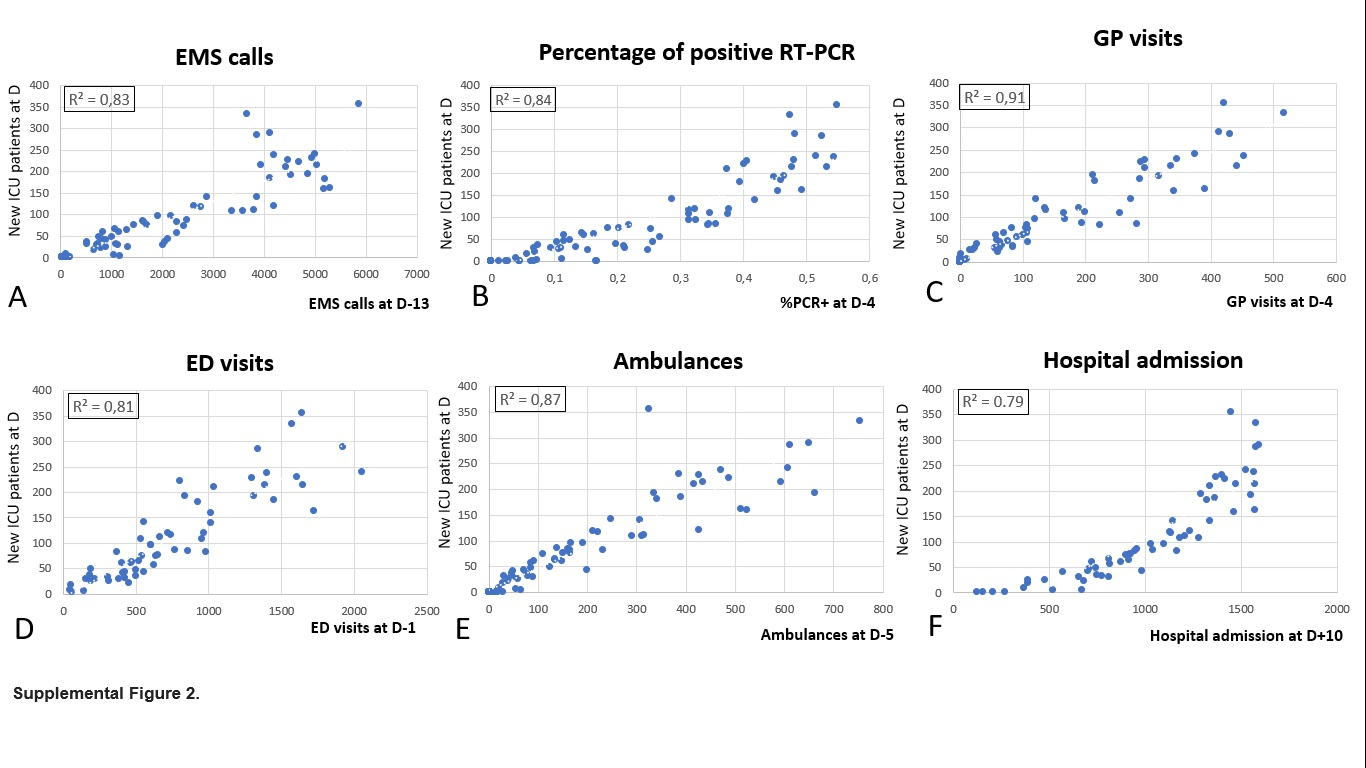

Supplement: S3 Fig — Correlation curves of the six tested indicators compared to the number of new intensive care unit (ICU) patients during the study period. EMS: emergency calls; GP: general practitioner; ED: emergency department. D: delay (in days) between the two variables. R2: Pearson coefficient of correlation. (TIF) [file pone.0241406.s003.tif]

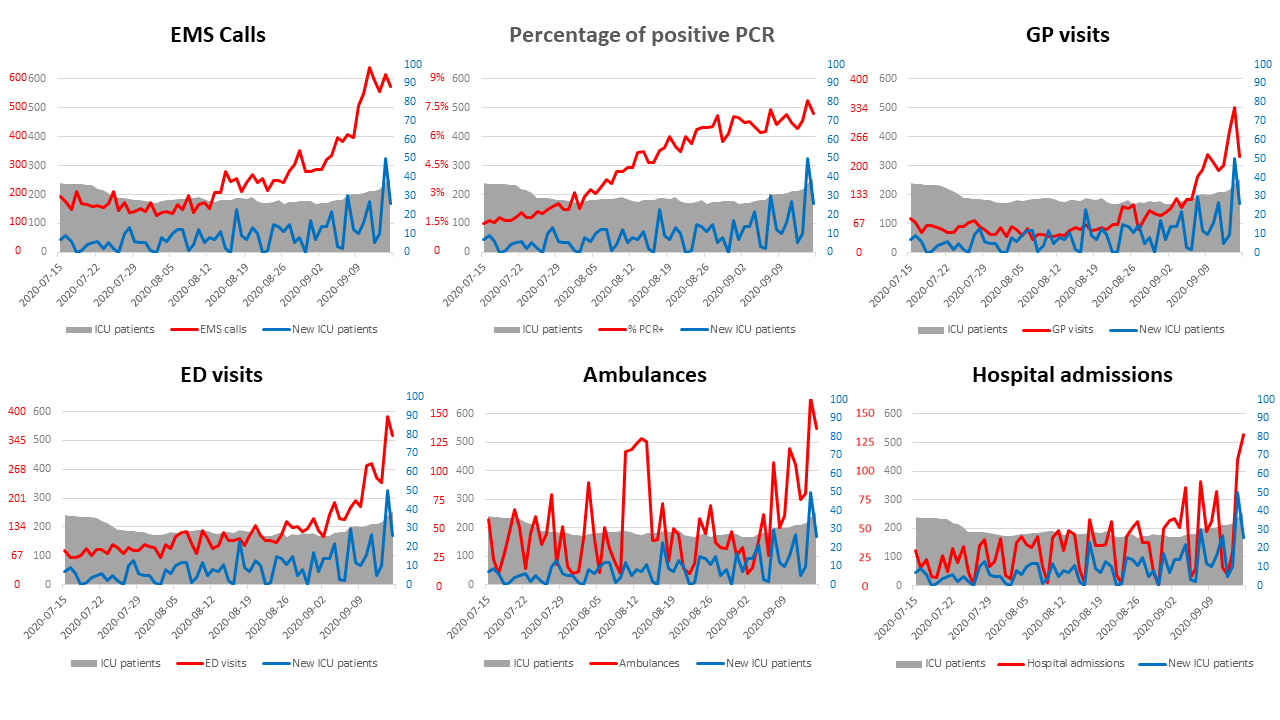

Supplement: S4 Fig — EMS: emergency calls; GP: general practitioner; ED: emergency department; RT-PCR: reverse transcriptase polymerase chain reaction tests. (TIF) [file pone.0241406.s004.tif]

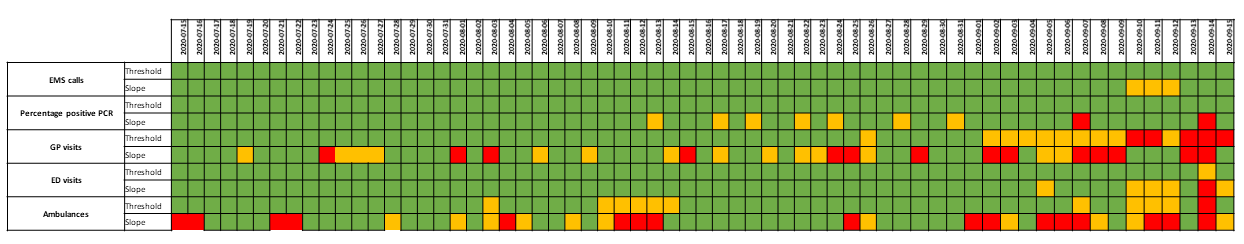

Supplement: S5 Fig — (TIF) [file pone.0241406.s005.tif]
