# Supplementary material for: Early indicators of intensive care unit bed requirement during the COVID-19 epidemic: A retrospective study in Ile-de-France region, France
Source: PLoS One. 2020 Nov 18;15(11):e0241406. doi: 10.1371/journal.pone.0241406 (PMC7673527; doi:10.1371/journal.pone.0241406)
Supplement: S1 Table — (DOCX) [file pone.0241406.s006.docx]

**S1 Table:** Number of emergency calls per day related to COVID-19 patients received by the four emergency medical system (EMS, i.e. SAMU) of the Assistance Publique-Hôpitaux de Paris (APHP) and number of these patients requiring dispatch of an ambulance (either ordinary or mobile intensive care unit (MICU)) from January 27 to September 15, 2020.

| **Date** | **EMS Calls/day** | **Ambulance/day** |
| --- | --- | --- |
| 2020-01-20 | 0 | 0 |
| 2020-01-21 | 0 | 0 |
| 2020-01-22 | 0 | 0 |
| 2020-01-23 | 0 | 0 |
| 2020-01-24 | 0 | 0 |
| 2020-01-25 | 0 | 0 |
| 2020-01-26 | 0 | 0 |
| 2020-01-27 | 180 | 5 |
| 2020-01-28 | 142 | 7 |
| 2020-01-29 | 160 | 3 |
| 2020-01-30 | 153 | 7 |
| 2020-01-31 | 179 | 11 |
| 2020-02-01 | 80 | 7 |
| 2020-02-02 | 80 | 4 |
| 2020-02-03 | 102 | 13 |
| 2020-02-04 | 61 | 3 |
| 2020-02-05 | 51 | 19 |
| 2020-02-06 | 63 | 5 |
| 2020-02-07 | 44 | 7 |
| 2020-02-08 | 59 | 6 |
| 2020-02-09 | 55 | 0 |
| 2020-02-10 | 94 | 2 |
| 2020-02-11 | 61 | 6 |
| 2020-02-12 | 65 | 7 |
| 2020-02-13 | 56 | 2 |
| 2020-02-14 | 50 | 3 |
| 2020-02-15 | 29 | 1 |
| 2020-02-16 | 26 | 1 |
| 2020-02-17 | 37 | 0 |
| 2020-02-18 | 40 | 2 |
| 2020-02-19 | 31 | 2 |
| 2020-02-20 | 30 | 0 |
| 2020-02-21 | 32 | 3 |
| 2020-02-22 | 33 | 1 |
| 2020-02-23 | 115 | 0 |
| 2020-02-24 | 659 | 1 |
| 2020-02-25 | 1179 | 9 |
| 2020-02-26 | 1063 | 13 |
| 2020-02-27 | 1133 | 8 |
| 2020-02-28 | 1327 | 17 |
| 2020-02-29 | 887 | 29 |
| 2020-03-01 | 887 | 15 |
| 2020-03-02 | 2019 | 21 |
| 2020-03-03 | 2063 | 30 |
| 2020-03-04 | 2301 | 65 |
| 2020-03-05 | 2434 | 56 |
| 2020-03-06 | 2481 | 49 |
| 2020-03-07 | 1628 | 60 |
| 2020-03-08 | 2292 | 41 |
| 2020-03-09 | 3589 | 49 |
| 2020-03-10 | 3870 | 91 |
| 2020-03-11 | 4113 | 77 |
| 2020-03-12 | 4947 | 86 |
| 2020-03-13 | 5872 | 110 |
| 2020-03-14 | 5043 | 139 |
| 2020-03-15 | 5009 | 162 |
| 2020-03-16 | 5308 | 233 |
| 2020-03-17 | 3942 | 312 |
| 2020-03-18 | 4125 | 308 |
| 2020-03-19 | 3677 | 391 |
| 2020-03-20 | 3869 | 387 |
| 2020-03-21 | 4195 | 325 |
| 2020-03-22 | 4538 | 435 |
| 2020-03-23 | 5172 | 608 |
| 2020-03-24 | 4688 | 513 |
| 2020-03-25 | 4481 | 593 |
| 2020-03-26 | 4442 | 650 |
| 2020-03-27 | 5197 | 755 |
| 2020-03-28 | 4870 | 612 |
| 2020-03-29 | 4191 | 472 |
| 2020-03-30 | 3810 | 663 |
| 2020-03-31 | 3363 | 525 |
| 2020-04-01 | 2886 | 488 |
| 2020-04-02 | 2755 | 428 |
| 2020-04-03 | 2631 | 417 |
| 2020-04-04 | 2165 | 341 |
| 2020-04-05 | 1909 | 335 |
| 2020-04-06 | 2113 | 428 |
| 2020-04-07 | 1637 | 315 |
| 2020-04-08 | 1701 | 291 |
| 2020-04-09 | 1443 | 249 |
| 2020-04-10 | 1311 | 222 |
| 2020-04-11 | 1153 | 212 |
| 2020-04-12 | 1079 | 191 |
| 2020-04-13 | 1101 | 167 |
| 2020-04-14 | 1010 | 199 |
| 2020-04-15 | 834 | 166 |
| 2020-04-16 | 764 | 151 |
| 2020-04-17 | 826 | 166 |
| 2020-04-18 | 807 | 134 |
| 2020-04-19 | 717 | 150 |
| 2020-04-20 | 735 | 135 |
| 2020-04-21 | 529 | 81 |
| 2020-04-22 | 518 | 124 |
| 2020-04-23 | 501 | 93 |
| 2020-04-24 | 492 | 86 |
| 2020-04-25 | 431 | 71 |
| 2020-04-26 | 298 | 39 |
| 2020-04-27 | 245 | 47 |
| 2020-04-28 | 277 | 47 |
| 2020-04-29 | 278 | 47 |
| 2020-04-30 | 242 | 32 |
| 2020-05-01 | 249 | 34 |
| 2020-05-02 | 293 | 57 |
| 2020-05-03 | 217 | 38 |
| 2020-05-04 | 203 | 25 |
| 2020-05-05 | 188 | 24 |
| 2020-05-06 | 210 | 40 |
| 2020-05-07 | 218 | 30 |
| 2020-05-08 | 307 | 40 |
| 2020-05-09 | 329 | 64 |
| 2020-05-10 | 258 | 55 |
| 2020-05-11 | 228 | 47 |
| 2020-05-12 | 191 | 51 |
| 2020-05-13 | 229 | 52 |
| 2020-05-14 | 207 | 27 |
| 2020-05-15 | 206 | 37 |
| 2020-05-16 | 200 | 31 |
| 2020-05-17 | 167 | 36 |
| 2020-05-18 | 191 | 44 |
| 2020-05-19 | 134 | 10 |
| 2020-05-20 | 193 | 13 |
| 2020-05-21 | 176 | 9 |
| 2020-05-22 | 165 | 51 |
| 2020-05-23 | 144 | 43 |
| 2020-05-24 | 111 | 6 |
| 2020-05-25 | 124 | 49 |
| 2020-05-26 | 94 | 20 |
| 2020-05-27 | 116 | 45 |
| 2020-05-28 | 118 | 10 |
| 2020-05-29 | 103 | 37 |
| 2020-05-30 | 120 | 33 |
| 2020-05-31 | 91 | 13 |
| 2020-06-01 | 97 | 31 |
| 2020-06-02 | 88 | 86 |
| 2020-06-03 | 86 | 44 |
| 2020-06-04 | 126 | 100 |
| 2020-06-05 | 82 | 44 |
| 2020-06-06 | 85 | 13 |
| 2020-06-07 | 75 | 18 |
| 2020-06-08 | 70 | 69 |
| 2020-06-09 | 50 | 13 |
| 2020-06-10 | 68 | 12 |
| 2020-06-11 | 60 | 44 |
| 2020-06-12 | 84 | 38 |
| 2020-06-13 | 88 | 15 |
| 2020-06-14 | 65 | 18 |
| 2020-06-15 | 60 | 70 |
| 2020-06-16 | 59 | 53 |
| 2020-06-17 | 61 | 13 |
| 2020-06-18 | 74 | 20 |
| 2020-06-19 | 64 | 19 |
| 2020-06-20 | 84 | 58 |
| 2020-06-21 | 66 | 10 |
| 2020-06-22 | 74 | 16 |
| 2020-06-23 | 79 | 41 |
| 2020-06-24 | 75 | 27 |
| 2020-06-25 | 67 | 55 |
| 2020-06-26 | 101 | 50 |
| 2020-06-27 | 116 | 11 |
| 2020-06-28 | 91 | 18 |
| 2020-06-29 | 104 | 27 |
| 2020-06-30 | 89 | 22 |
| 2020-07-01 | 127 | 34 |
| 2020-07-02 | 93 | 12 |
| 2020-07-03 | 109 | 32 |
| 2020-07-04 | 125 | 20 |
| 2020-07-05 | 109 | 32 |
| 2020-07-06 | 119 | 43 |
| 2020-07-07 | 108 | 20 |
| 2020-07-08 | 138 | 10 |
| 2020-07-09 | 140 | 29 |
| 2020-07-10 | 164 | 19 |
| 2020-07-11 | 152 | 15 |
| 2020-07-12 | 132 | 40 |
| 2020-07-13 | 178 | 45 |
| 2020-07-14 | 214 | 19 |
| 2020-07-15 | 192 | 57 |
| 2020-07-16 | 175 | 22 |
| 2020-07-17 | 149 | 10 |
| 2020-07-18 | 209 | 28 |
| 2020-07-19 | 168 | 47 |
| 2020-07-20 | 167 | 66 |
| 2020-07-21 | 159 | 50 |
| 2020-07-22 | 161 | 14 |
| 2020-07-23 | 154 | 45 |
| 2020-07-24 | 169 | 60 |
| 2020-07-25 | 210 | 35 |
| 2020-07-26 | 146 | 45 |
| 2020-07-27 | 173 | 79 |
| 2020-07-28 | 137 | 18 |
| 2020-07-29 | 143 | 51 |
| 2020-07-30 | 152 | 16 |
| 2020-07-31 | 143 | 10 |
| 2020-08-01 | 171 | 12 |
| 2020-08-02 | 128 | 41 |
| 2020-08-03 | 137 | 90 |
| 2020-08-04 | 142 | 40 |
| 2020-08-05 | 134 | 11 |
| 2020-08-06 | 165 | 50 |
| 2020-08-07 | 147 | 32 |
| 2020-08-08 | 195 | 19 |
| 2020-08-09 | 137 | 10 |
| 2020-08-10 | 167 | 117 |
| 2020-08-11 | 172 | 119 |
| 2020-08-12 | 153 | 124 |
| 2020-08-13 | 206 | 128 |
| 2020-08-14 | 206 | 126 |
| 2020-08-15 | 278 | 39 |
| 2020-08-16 | 245 | 40 |
| 2020-08-17 | 257 | 71 |
| 2020-08-18 | 209 | 20 |
| 2020-08-19 | 240 | 49 |
| 2020-08-20 | 270 | 44 |
| 2020-08-21 | 241 | 14 |
| 2020-08-22 | 256 | 10 |
| 2020-08-23 | 213 | 19 |
| 2020-08-24 | 247 | 58 |
| 2020-08-25 | 250 | 45 |
| 2020-08-26 | 241 | 17 |
| 2020-08-27 | 283 | 37 |
| 2020-08-28 | 304 | 33 |
| 2020-08-29 | 352 | 32 |
| 2020-08-30 | 279 | 47 |
| 2020-08-31 | 281 | 26 |
| 2020-09-01 | 288 | 33 |
| 2020-09-02 | 285 | 10 |
| 2020-09-03 | 322 | 15 |
| 2020-09-04 | 335 | 39 |
| 2020-09-05 | 396 | 62 |
| 2020-09-06 | 382 | 26 |
| 2020-09-07 | 408 | 107 |
| 2020-09-08 | 398 | 50 |
| 2020-09-09 | 509 | 61 |
| 2020-09-10 | 550 | 120 |
| 2020-09-11 | 638 | 106 |
| 2020-09-12 | 595 | 75 |
| 2020-09-13 | 555 | 80 |
| 2020-09-14 | 615 | 164 |
| 2020-09-15 | 573 | 137 |
