# Supplementary material for: Early indicators of intensive care unit bed requirement during the COVID-19 epidemic: A retrospective study in Ile-de-France region, France
Source: PLoS One. 2020 Nov 18;15(11):e0241406. doi: 10.1371/journal.pone.0241406 (PMC7673527; doi:10.1371/journal.pone.0241406)
Supplement: S2 Table — (DOCX) [file pone.0241406.s007.docx]

**S2 Table.** Median daily number of emergency calls received by the emergency medical system, ambulances sent, general practitioner (GP) visits, emergency department (ED) visits, reverse transcriptase polymerase chain transmission (RT-PCR), and hospital admission in al; patients and COVID-19 patients during the study period.

| **Variables** | **All patients**  Daily number [IQR] | **Covid-19 Patients**  Extremes (%*) |
| --- | --- | --- |
| Emergency calls | 1536 [494-3854] | 0 to 5872 (0-66%) |
| Ambulances | 48 [8·5-173] | 0 to 354 (0-47%) |
| GP visits | 91 [56-255] | 0 to 518 (0-30%) |
| ED visits | 519 [211-954] | 0 to 2054 (0-37%) |
| RT-PCR | 1301 [600-3724] | 0 to 4162 (0-54 %) |
| Hospital admission | 5552 [1148-7533] | 0 to 13450 (0-NA) |

*: the maximum of the percentage may not correspond to the maximum of number.

NA: not available
