# Supplementary material for: Early indicators of intensive care unit bed requirement during the COVID-19 epidemic: A retrospective study in Ile-de-France region, France
Source: PLoS One. 2020 Nov 18;15(11):e0241406. doi: 10.1371/journal.pone.0241406 (PMC7673527; doi:10.1371/journal.pone.0241406)
Supplement: Peer review history

# Peer review: Decision Letter 0

**DOI:** 10.1371/journal.pone.0241406.r001

**Authors:** Corstiaan den Uil

16 Sep 2020

PONE-D-20-23904

Emergency calls are early indicators of ICU bed requirement during the COVID-19 epidemic. 

A retrospective study in Ile-de-France region, France

PLOS ONE

Dear Dr. RIOU,

Thank you for submitting your manuscript to PLOS ONE. After careful consideration, we feel that it has merit but does not fully meet PLOS ONE’s publication criteria as it currently stands. Therefore, we invite you to submit a revised version of the manuscript that addresses the points raised during the review process.

Please submit your revised manuscript by Oct 31 2020 11:59PM. If you will need more time than this to complete your revisions, please reply to this message or contact the journal office at plosone@plos.org. When you're ready to submit your revision, log on to https://www.editorialmanager.com/pone/ and select the 'Submissions Needing Revision' folder to locate your manuscript file.

Please include the following items when submitting your revised manuscript:

- A rebuttal letter that responds to each point raised by the academic editor and reviewer(s). You should upload this letter as a separate file labeled 'Response to Reviewers'.
- A marked-up copy of your manuscript that highlights changes made to the original version. You should upload this as a separate file labeled 'Revised Manuscript with Track Changes'.
- An unmarked version of your revised paper without tracked changes. You should upload this as a separate file labeled 'Manuscript'.

If you would like to make changes to your financial disclosure, please include your updated statement in your cover letter. Guidelines for resubmitting your figure files are available below the reviewer comments at the end of this letter.

If applicable, we recommend that you deposit your laboratory protocols in protocols.io to enhance the reproducibility of your results. Protocols.io assigns your protocol its own identifier (DOI) so that it can be cited independently in the future. For instructions see: http://journals.plos.org/plosone/s/submission-guidelines#loc-laboratory-protocols

We look forward to receiving your revised manuscript.

Kind regards,

Corstiaan den Uil

Academic Editor

PLOS ONE

Journal Requirements:

When submitting your revision, we need you to address these additional requirements.

1. Please ensure that your manuscript meets PLOS ONE's style requirements, including those for file naming. The PLOS ONE style templates can be found at

https://journals.plos.org/plosone/s/file?id=wjVg/PLOSOne_formatting_sample_main_body.pdf and

https://journals.plos.org/plosone/s/file?id=ba62/PLOSOne_formatting_sample_title_authors_affiliations.pdf

2. We note that in your Ethics Statement you have provided information that your Ethics Approval committee waived the need for informed patient consent, as data were anonymized.

Please amend you Methods section to also include this information.

3. We note that you have indicated that data from this study are available upon request. PLOS only allows data to be available upon request if there are legal or ethical restrictions on sharing data publicly. For information on unacceptable data access restrictions, please see http://journals.plos.org/plosone/s/data-availability#loc-unacceptable-data-access-restrictions.

In your revised cover letter, please address the following prompts:

a) If there are ethical or legal restrictions on sharing a de-identified data set, please explain them in detail (e.g., data contain potentially identifying or sensitive patient information) and who has imposed them (e.g., an ethics committee). Please also provide contact information for a data access committee, ethics committee, or other institutional body to which data requests may be sent.

b) If there are no restrictions, please upload the minimal anonymized data set necessary to replicate your study findings as either Supporting Information files or to a stable, public repository and provide us with the relevant URLs, DOIs, or accession numbers. Please see http://www.bmj.com/content/340/bmj.c181.long for guidelines on how to de-identify and prepare clinical data for publication. For a list of acceptable repositories, please see http://journals.plos.org/plosone/s/data-availability#loc-recommended-repositories.

We will update your Data Availability statement on your behalf to reflect the information you provide.

4. Thank you for stating the following financial disclosure:

'No. The funders had no role in study design, data collection and analysis, decision to publish, or preparation of the manuscript.'

At this time, please address the following queries:

1. Please clarify the sources of funding (financial or material support) for your study. List the grants or organizations that supported your study, including funding received from your institution.
2. State what role the funders took in the study. If the funders had no role in your study, please state: “The funders had no role in study design, data collection and analysis, decision to publish, or preparation of the manuscript.”
3. If any authors received a salary from any of your funders, please state which authors and which funders.
4. If you did not receive any funding for this study, please state: “The authors received no specific funding for this work.”

Please include your amended statements within your cover letter; we will change the online submission form on your behalf.

5. Your ethics statement should only appear in the Methods section of your manuscript. If your ethics statement is written in any section besides the Methods, please delete it from any other section.

6. Please include captions for your Supporting Information files at the end of your manuscript, and update any in-text citations to match accordingly. Please see our Supporting Information guidelines for more information: http://journals.plos.org/plosone/s/supporting-information

[Note: HTML markup is below. Please do not edit.]

Reviewers' comments:

Reviewer's Responses to Questions

**Comments to the Author**

1. Is the manuscript technically sound, and do the data support the conclusions?

The manuscript must describe a technically sound piece of scientific research with data that supports the conclusions. Experiments must have been conducted rigorously, with appropriate controls, replication, and sample sizes. The conclusions must be drawn appropriately based on the data presented. 

Reviewer #1: Partly

Reviewer #2: Partly

**********

2. Has the statistical analysis been performed appropriately and rigorously? 

Reviewer #1: I Don't Know

Reviewer #2: Yes

**********

3. Have the authors made all data underlying the findings in their manuscript fully available?

The PLOS Data policy requires authors to make all data underlying the findings described in their manuscript fully available without restriction, with rare exception (please refer to the Data Availability Statement in the manuscript PDF file). The data should be provided as part of the manuscript or its supporting information, or deposited to a public repository. For example, in addition to summary statistics, the data points behind means, medians and variance measures should be available. If there are restrictions on publicly sharing data—e.g. participant privacy or use of data from a third party—those must be specified.

Reviewer #1: Yes

Reviewer #2: Yes

**********

4. Is the manuscript presented in an intelligible fashion and written in standard English?

PLOS ONE does not copyedit accepted manuscripts, so the language in submitted articles must be clear, correct, and unambiguous. Any typographical or grammatical errors should be corrected at revision, so please note any specific errors here.

Reviewer #1: Yes

Reviewer #2: No

**********

5. Review Comments to the Author

Please use the space provided to explain your answers to the questions above. You may also include additional comments for the author, including concerns about dual publication, research ethics, or publication ethics. (Please upload your review as an attachment if it exceeds 20,000 characters)

Reviewer #1: Summary of the manuscript:

The authors retrospectively investigated predictors for ICU capacity needs due to COVID-19 in the Ile-de-France region, France, between February 20 and May 5, 2020. Indicators that were found to be correlated with ICU needs (with a delay) included EMS calls, percentage of positive RT-PCR tests, GP visits, ED visits and ambulances.

This is an extremely important question for the preparation of a potential second wave of COVID-19 in the region, and the authors have argued its importance well in the introduction. However, I feel the conclusions could have been much stronger had authors treated this research question as a prediction problem, and use appropriate prediction methods to address this problem. Treating this as a prediction problem and validate the prediction tool in an external sample would help with generalizing the result to the future and inform practice. Please see my major comments below.

Major comments and suggestions to the authors:

1. The correlation between many indicators and the outcomes may not be generalizable to the future (which is the main goal of this paper). For example, the authors used % test positive as one indicator, but % test positive, although an indicator for the spread of SARS-CoV-2 in the community during the early phases of the pandemic (due to testing shortage), in many settings it is starting to become an indicator of testing availability and how widespread testing was, rather than an indicator for community spread. Even if % test positive could indicate ICU capacity needs in the early phases of the pandemic in the Ile-de-France regioon, its correlation with ICU needs is unlikely to stay the same during a second wave of COVID-19. Similar arguments can be made for other indicators as well, where policy and resource availability changes can potentially change the correlation between an indicator and the outcome.

• Suggestion: use an external validation dataset (either another time frame, or data from another region) to validate any prediction rules the authors concluded. For example, the authors concluded that EMS calls is the best early indicators for covid-19 ICU needs – does this hold for another region? And does it hold in the same region, but with more recent data?

2. It is unclear why the authors used univariate indicators, rather than combining some indicators into a prediction model which could potentially better predict the outcome.

• Suggestion: consider combining predictors into a prediction model. It is very likely that combining the included indicators would yield the best prediction. Alternatively, discuss why using a single indicator is the best approach here (for ease of use?)

3. Suggestion: please expand the first paragraph on page 7 – in its current form I cannot understand the main portion of the analysis (and Table 1) so it is hard to assess the validity of the general method.

4. As it is currently being described, I don’t think using the indicators to estimate a curve for R(t) is a valid approach, nor do I think it is useful for the overall purpose of this paper.

• Suggestion: I suggest the authors exclude reporting this analysis and focus on the actual prediction of ICU use.

5. Overall suggestion on methods: Reframing the question as a prediction problem and follow standard reporting criteria for reporting prediction models, e.g. https://www.equator-network.org/reporting-guidelines/tripod-statement/

6. The authors mentioned that “massive inter-regional ICU patient transfers” took place to ensure all patients requiring ICU were admitted. However, it is unclear whether this transfer of ICU patients was captured in the data and how the authors had accounted for this. If assuming that the Ile-de-France region had a surplus of ICU patients during the peak of the first wave, that means many patients were transferred to other regions to be treated. Does this mean that the outcome data used in this analysis is an underestimation of ICU patients at the peak of the epidemic?

a. Suggestion: please explain how the transfer of ICU patients influence (or not influence) the interpretation of data in your analysis.

Minor comment:

7. Please put the first paragraph of Results into a table for ease of reading. In particular, it is unclear why the authors chose to report median and IQR for overall measures, and range for COVID-specific measures.

Reviewer #2: The authors presented the results or their investigation describing the response to COVID-19 in some regions of France and tried to find any relationship among the emergency calls and ICU beds occupation. The topic is very interesting and intriguing with the perspective of improving hospital surge capacity response to COVID-19 patients. Unfortunately, the paper needs an extensive english language editing because, in same case, it is very difficult to understand. Following my specific comments

Abstract

1) no data and p values referring to correlation analysis are reported in the abstract.

Paper

Introduction

1) Main aim: in its actual form the main object of the study is not clear. Please rephrase starting from your hypothesis. clarify your hypothesis by pointing out that some indicators ( as the number of telephone calls seems to be) may help in predicting the hospital and ICU surge capacity crisis, for example. This is the pivotal element of the study and, in my opinion, it should be better constructed and argued than actually is.

Methods

2) it seems that the response to an emergency call purely depend on operator judgment. Do you know whether any clinical protocol is employed to manage emergency call? if yes, i think it should be reported in the text.

3) statistical analysis "For each indicator, we determined the onset defined as the first day the indicator became

positive" what does it mean that indicators becomes positive ?

4) "We performed correlation curve analysis during the whole study period by plotting (ICU patients at date T) vs (value of the indicator at date T+t) and varying t, to determine the best correlation coefficient, depending on the number of

days the indicator had been shifted. Please, explain T and t what are referred.

Results

5) "Figure 1 shows the comparison of each indicator to the primary and secondary endpoints". you are referring to the title of a table and you should describe what primary and secondary endpoint mean.

**********

6. PLOS authors have the option to publish the peer review history of their article (what does this mean?). If published, this will include your full peer review and any attached files.

If you choose “no”, your identity will remain anonymous but your review may still be made public.

**Do you want your identity to be public for this peer review?** For information about this choice, including consent withdrawal, please see our Privacy Policy.

Reviewer #1: No

Reviewer #2: No

[NOTE: If reviewer comments were submitted as an attachment file, they will be attached to this email and accessible via the submission site. Please log into your account, locate the manuscript record, and check for the action link "View Attachments". If this link does not appear, there are no attachment files.]

While revising your submission, please upload your figure files to the Preflight Analysis and Conversion Engine (PACE) digital diagnostic tool, https://pacev2.apexcovantage.com/. PACE helps ensure that figures meet PLOS requirements. To use PACE, you must first register as a user. Registration is free. Then, login and navigate to the UPLOAD tab, where you will find detailed instructions on how to use the tool. If you encounter any issues or have any questions when using PACE, please email PLOS at figures@plos.org. Please note that Supporting Information files do not need this step.

# Author comment: Author response to Decision Letter 0

**DOI:** 10.1371/journal.pone.0241406.r002

9 Oct 2020

RESPONSE TO THE EDITOR AND REVIEWERS

We thank the Editor and the reviewers for their helpful comments. We would like to offer the following responses to the comments from the Editor and Reviewers and hope that our manuscript will be suitable for publication in PLoS ONE.

EDITOR’S COMMENTS

1. Please ensure that your manuscript meets PLOS ONE's style requirements, including those for file naming. The PLOS ONE style templates can be found at

https://clicktime.symantec.com/3M61Pxxnm2n162yZGsrzHFG6H2?u=https%3A%2F%2Fjournals.plos.org%2Fplosone%2Fs%2Ffile%3Fid%3DwjVg%2FPLOSOne_formatting_sample_main_body.pdf and

https://clicktime.symantec.com/3VT65YhNMGf95WGgVQNpJdX6H2?u=https%3A%2F%2Fjournals.plos.org%2Fplosone%2Fs%2Ffile%3Fid%3Dba62%2FPLOSOne_formatting_sample_title_authors_affiliations.pdf

Response: We have taken into consideration all indications provided. All changes have been underlined in yellow in the “Manuscript with tracked changes”. 

2. We note that in your Ethics Statement you have provided information that your Ethics Approval committee waived the need for informed patient consent, as data were anonymized. Please amend you Methods section to also include this information.

Response: Correction performed in the revised version (Page 5, line 3) 

3. We note that you have indicated that data from this study are available upon request. PLOS only allows data to be available upon request if there are legal or ethical restrictions on sharing data publicly. For information on unacceptable data access restrictions, please see https://clicktime.symantec.com/3Un5xxLxtXkZvx31VC4hhoA6H2?u=http%3A%2F%2Fjournals.plos.org%2Fplosone%2Fs%2Fdata-availability%23loc-unacceptable-data-access-restrictions.

In your revised cover letter, please address the following prompts:

a) If there are ethical or legal restrictions on sharing a de-identified data set, please explain them in detail (e.g., data contain potentially identifying or sensitive patient information) and who has imposed them (e.g., an ethics committee). Please also provide contact information for a data access committee, ethics committee, or other institutional body to which data requests may be sent.

b) If there are no restrictions, please upload the minimal anonymized data set necessary to replicate your study findings as either Supporting Information files or to a stable, public repository and provide us with the relevant URLs, DOIs, or accession numbers. Please see https://clicktime.symantec.com/34KLqYb4t8uy2wmTaXz5Fj46H2?u=http%3A%2F%2Fwww.bmj.com%2Fcontent%2F340%2Fbmj.c181.long for guidelines on how to de-identify and prepare clinical data for publication. For a list of acceptable repositories, please see https://clicktime.symantec.com/3843Rhus5YdvdNf4zA5eu586H2?u=http%3A%2F%2Fjournals.plos.org%2Fplosone%2Fs%2Fdata-availability%23loc-recommended-repositories.

We will update your Data Availability statement on your behalf to reflect the information you provide.

Response: Some of the raw data (number of general practitioner and emergency department visits, percentage of RT-PCR, number of ICU patients, number of hospitalizations) are publicly available (https://www.data.gouv.fr) and this information is clearly provided in the method section. Other raw data not publicly available (EMS calls and ambulance dispatches) are now provided as supplement information (S1 Table). We think that we now follow PLoS ONE policy concerning data availability. 

4. Thank you for stating the following financial disclosure:

'No. The funders had no role in study design, data collection and analysis, decision to publish, or preparation of the manuscript.'

At this time, please address the following queries:

a. Please clarify the sources of funding (financial or material support) for your study. List the grants or organizations that supported your study, including funding received from your institution.

b. State what role the funders took in the study. If the funders had no role in your study, please state: “The funders had no role in study design, data collection and analysis, decision to publish, or preparation of the manuscript.”

c. If any authors received a salary from any of your funders, please state which authors and which funders.

d. If you did not receive any funding for this study, please state: “The authors received no specific funding for this work.”

Please include your amended statements within your cover letter; we will change the online submission form on your behalf.

Response: In the revised version, we have added the following statement: “The funders had no role in study design, data collection and analysis, decision to publish, or preparation of the manuscript. However, one member of the authors’ consortium is the Chief Executive Officer of one of these institutions, namely Martin Hirsch for Assistance Publique-Hôpitaux de Paris.” (Page 20, lines 15-18).

5. Your ethics statement should only appear in the Methods section of your manuscript. If your ethics statement is written in any section besides the Methods, please delete it from any other section.

Response: In the revised version, we have deleted the two sections at the end of the text which were named “Ethics approval and consent to participate” and “Consent for publication”

6. Please include captions for your Supporting Information files at the end of your manuscript, and update any in-text citations to match accordingly. Please see our Supporting Information guidelines for more information: http://journals.plos.org/plosone/s/supporting-information

Response: Supporting information has been provided the end of the manuscript and in-text citations have been corrected.

7. While revising your submission, please upload your figure files to the Preflight Analysis and Conversion Engine (PACE) digital diagnostic tool, https://pacev2.apexcovantage.com/. PACE helps ensure that figures meet PLOS requirements. To use PACE, you must first register as a user. Registration is free. Then, login and navigate to the UPLOAD tab, where you will find detailed instructions on how to use the tool. If you encounter any issues or have any questions when using PACE, please email PLOS at figures@plos.org. Please note that Supporting Information files do not need this step.

Response: We have used the PACE digital diagnostic tool and provided in the revised version only figures that meet PLOS requirements. Thus, we have slightly modified Figure 3 in order it meet this requirements. 

REVIEWER #1

The authors retrospectively investigated predictors for ICU capacity needs due to COVID-19 in the Ile-de-France region, France, between February 20 and May 5, 2020. Indicators that were found to be correlated with ICU needs (with a delay) included EMS calls, percentage of positive RT-PCR tests, GP visits, ED visits and ambulances.

This is an extremely important question for the preparation of a potential second wave of COVID-19 in the region, and the authors have argued its importance well in the introduction. However, I feel the conclusions could have been much stronger had authors treated this research question as a prediction problem, and use appropriate prediction methods to address this problem. Treating this as a prediction problem and validate the prediction tool in an external sample would help with generalizing the result to the future and inform practice. Please see my major comments below.

1. The correlation between many indicators and the outcomes may not be generalizable to the future (which is the main goal of this paper). For example, the authors used % test positive as one indicator, but % test positive, although an indicator for the spread of SARS-CoV-2 in the community during the early phases of the pandemic (due to testing shortage), in many settings it is starting to become an indicator of testing availability and how widespread testing was, rather than an indicator for community spread. Even if % test positive could indicate ICU capacity needs in the early phases of the pandemic in the Ile-de-France region, its correlation with ICU needs is unlikely to stay the same during a second wave of COVID-19. Similar arguments can be made for other indicators as well, where policy and resource availability changes can potentially change the correlation between an indicator and the outcome.

• Suggestion: use an external validation dataset (either another time frame, or data from another region) to validate any prediction rules the authors concluded. For example, the authors concluded that EMS calls is the best early indicators for covid-19 ICU needs – does this hold for another region? And does it hold in the same region, but with more recent data?

Response: We thank the Reviewer for this important comment. We have tried to avoid as much as possible the word and concept of “prediction” because we think that our study cannot really predict the evolution of the epidemic itself for many reasons clearly explained to the reader in the introduction when we said that “The peak of the crisis depends indeed on collective measures (testing, isolation of infected patients, social distancing, wearing mask, hand washing, and lockdown), which are the only actions with proven efficacy in the absence of proven specific treatment and/or vaccination to date [9]” (Page 3, lines 13-16). In contrast, we think that our study only provides an anticipated estimation of the number of ICU beds required by analysing variables that are obviously time-linked to it, with different time lag. Concerning the methodology of prediction applied to this problem, we have tried to apply the TRIPOD criteria as much as possible but many of them do not apply to this study, particularly performance reporting, blinding, and use of multivariate analysis (see our response to comment ≠ 2 below). However, the main criticism of the Reviewer is the lack of external validation in our study and we must recognize that this is an important criticism. Unfortunately we were unable to conduct such analyses in other regions of France because only two of them were markedly involved in the epidemic during the first wave and, overall, we should be unable to obtain comparable data for all indicators tested. It should be noted that the computer systems in EMS are very heterogeneous in France and the identification of emergency calls for Covid-19 has not been done in the same reliable way we used. Thus it is not possible for us to provide external “geographical” validation. At the moment of submission of this manuscript (July) we were also unable to assess external validity by using another time frame which did not yet exist. This is clearly not the case now because our region (in fact the whole France as for many other countries) is facing what we think is the onset of a second epidemic wave. Therefore, we have conducted a new analysis during this second period (From August 1 to September 15, 2020) and provide the corresponding qualitative analysis in the revised version which provided similar results. Thus, in the revised version, we have added a sentence in the abstract (Page 2, lines 15-17), in the methods section (Page 7, lines 1-4), and in the discussion (Page 13, lines 21-23) and added new figures as supplement information (Figs S4 and S5). Only a qualitative analysis could be performed since data on primary and secondary endpoints (ICU patients) are obviously truncated during this very early phase and thus anticipation delays could not be calculated. We hope that will alleviate the Reviewer’s criticism since these new results confirm our initial hypothesis. 

2. It is unclear why the authors used univariate indicators, rather than combining some indicators into a prediction model which could potentially better predict the outcome.

• Suggestion: consider combining predictors into a prediction model. It is very likely that combining the included indicators would yield the best prediction. Alternatively, discuss why using a single indicator is the best approach here (for ease of use?)

Response: We did not want to perform a multivariate analysis for several reasons. First, we think that some of these indicators may not be available in some countries or regions, precluding the use of a multivariate indicator elsewhere. Second, an important result of the analysis is the time delay between each indicator and the endpoint (ICU patients). Using a multivariate indicator may provide more robust indications, but will be less timely than the earliest components. In contrast, having several indicators with different time delays may provide a series of successive alerts which reinforce each other. This is really what happened and how we dealt on a daily basis with this panel of indicators just before the occurrence of the second wave. However, we think that the comment of the Reviewer deserve additional information for the reader to explain why a univariate analysis has been preferred. Thus, this point has been added in the revised version (Page 12, lines 15-18). We thank the Reviewer for helping us to better explain that important issue to the reader. 

3. Suggestion: please expand the first paragraph on page 7 – in its current form I cannot understand the main portion of the analysis (and Table 1) so it is hard to assess the validity of the general method.

Response: In the revised version, we have tried to better describe the methods used (Page 7, lines 9-10 ). T is clearly defined as the date and t is now defined as the time lag expressed as number of days).

4. As it is currently being described, I don’t think using the indicators to estimate a curve for R(t) is a valid approach, nor do I think it is useful for the overall purpose of this paper.

• Suggestion: I suggest the authors exclude reporting this analysis and focus on the actual prediction of ICU use.

Response: We respectfully disagree with the Reviewer. All indicators included in our study reflect the epidemic curve, obviously to a scaling factor and with a lag. The computation of R(t) therefore provides a valid estimate of the reproduction ratio of the epidemic, as long as the relation between incidence and the indicators remains the same. Here we show that all indicators are likely to reflect the number of Covid-19 cases, as the time-dependant reproduction ratio R(t) computed from these different sources of information yield very commensurate estimates, taking into account the time delay between them. We think that this figure is important to convince epidemiologists that these variables should be considered as variables which appropriately and accurately described the evolution of the epidemic (Page 9, lines 20-21). This point has been added in the revised version to better explain our purpose to the reader (Page 7, lines 11-12). 

5. Overall suggestion on methods: Reframing the question as a prediction problem and follow standard reporting criteria for reporting prediction models, e.g. https://clicktime.symantec.com/3GC2PiatM8PM9sHgF7EkmUW6H2?u=https%3A%2F%2Fwww.equator-network.org%2Freporting-guidelines%2Ftripod-statement%2F

Response : As indicated in our response to comment ≠1 above, we wish to avoid the word and concept of prediction in the text of the manuscript. Nevertheless, in the revised version, we have tried to apply the TRIPOD recommendations, at least for the applicable criteria.

6. The authors mentioned that “massive inter-regional ICU patient transfers” took place to ensure all patients requiring ICU were admitted. However, it is unclear whether this transfer of ICU patients was captured in the data and how the authors had accounted for this. If assuming that the Ile-de-France region had a surplus of ICU patients during the peak of the first wave, that means many patients were transferred to other regions to be treated. Does this mean that the outcome data used in this analysis is an underestimation of ICU patients at the peak of the epidemic?

a. Suggestion: please explain how the transfer of ICU patients influence (or not influence) the interpretation of data in your analysis.

Response: The Reviewer is correct. In this study, we did not take into account the interregional transfers because they occurred only during the very last period. Moreover, the term “massive” was used because these transfers were unprecedented in France (n=349, including 164 to other countries, i.e. international transfers to Germany, Switzerland, Austria, and Luxembourg but these international transfer did not involve our Ile-de-France region) and because they required considerable human and logistic efforts (fixed wing aircraft, helicopters, and high-speed train) and concerned only critically ill patients requiring mechanical ventilations. However, the number of transferred patients in our region were 252 and represents only 3.9 % of all critically ill patients admitted in ICU. Moreover, concerning the patients transferred to other regions, only part of their length of stay was concerned and since no patient was transferred during the early days after admission into ICU, this issue have no impact on the secondary endpoint (new patients in ICI). In the revised version, to take into account the important comment of the Reviewer, we now clearly indicate the proportion of patients transferred (Page 9, lines 8-10) and discussed this limitation in the discussion (Page 14, lines 14-15 ). In the revised version, we have also indicated that in the method section (Page 5, line 25). We also deleted the term “massive”.

7. Please put the first paragraph of Results into a table for ease of reading. In particular, it is unclear why the authors chose to report median and IQR for overall measures, and range for COVID-specific measures.

Response: We think that these data are important for the reader to understand the amplitude of variation of each variables and for future comparisons with other regions or countries and thus we wish to maintain them. Nevertheless, we agree that this information may not be included in the main text and that a table might be easier to read. In the revised version, we have provided this information as a supportive information (S1 table, Page 26). We hope that this proposition will satisfy both the Reviewer and the Editor. 

REVIEWER #2: 

The authors presented the results or their investigation describing the response to COVID-19 in some regions of France and tried to find any relationship among the emergency calls and ICU beds occupation. The topic is very interesting and intriguing with the perspective of improving hospital surge capacity response to COVID-19 patients. Unfortunately, the paper needs an extensive english language editing because, in same case, it is very difficult to understand. Following my specific comments.

1.Abstract: no data and p values referring to correlation analysis are reported in the abstract.

Response: In the revised version, we have indicated the range of coefficient of correlation and the P values (all P<0.001) in the abstract (Page 2, line 13).

2.Paper. Introduction. Main aim: in its actual form the main object of the study is not clear. Please rephrase starting from your hypothesis. clarify your hypothesis by pointing out that some indicators ( as the number of telephone calls seems to be) may help in predicting the hospital and ICU surge capacity crisis, for example. This is the pivotal element of the study and, in my opinion, it should be better constructed and argued than actually is.

Response: We did not see how to better present our introduction which is brief and clearly present the background of the need to anticipate ICU beds, the lack of information from the literature, the aim of the study and the alert signal studied, and lastly why this could be important for health authorities. 

3. Methods: it seems that the response to an emergency call purely depend on operator judgment. Do you know whether any clinical protocol is employed to manage emergency call? if yes, I think it should be reported in the text.

Response: Yes, a clinical protocol was employed to manage emergency calls, according to the Ministry of Health recommendations. This information has been added in the revised version (Page 6, lines 11-12) with a citation of the appropriate reference (new reference ≠14). 

4. Method. Statistical analysis "For each indicator, we determined the onset defined as the first day the indicator became positive" what does it mean that indicators becomes positive ?

Response: This means that the indicator is greater than zero. This has now been precisely indicated in the revised version (Page 7, line 3). 

5. Method. "We performed correlation curve analysis during the whole study period by plotting (ICU patients at date T) vs (value of the indicator at date T+t) and varying t, to determine the best correlation coefficient, depending on the number of

days the indicator had been shifted. Please, explain T and t what are referred.

Response: In the revised version, we have tried to better describe the methods used (Page 7, lines 9-10 ). T is clearly defined as the date and t is now defined as the time lag expressed as number of days).

6. Results: "Figure 1 shows the comparison of each indicator to the primary and secondary endpoints". you are referring to the title of a table and you should describe what primary and secondary endpoint mean.

Response: Correction performed in the revised version (Page 8, lines 9-10).

Other changes performed:

1. Since the preprint cited as a footnote in the text has been accepted for publication, we have included it in the reference list as reference ≠ 33.

2. Because of inclusion of a new reference in the revised version (≠14) all subsequent references have been re-numbered.

3. We changed the title from “Emergency calls are early indicators of intensive care unit bed requirement during the Covid-19 epidemic” to “Early indicators of intensive care unit bed requirement during the Covid-19 epidemic” for two main reasons: a) this title more appropriately reflects the conclusion in the text and in the abstract since it did not focus only on EMS calls; b) we think that this modification is appropriate considering our response to comment ≠2 from Reviewer ≠1.

Attachment Submitted filename: RESPONSE TO THE EDITOR AND REVIEWERS.docx Click here for additional data file.

# Editor report: Decision Letter 1

**DOI:** 10.1371/journal.pone.0241406.r003

**Authors:** Corstiaan den Uil

15 Oct 2020

Early indicators of intensive care unit bed requirement

during the Covid-19 epidemic

A retrospective study in Ile-de-France region, France

PONE-D-20-23904R1

Dear Dr. RIOU,

We’re pleased to inform you that your manuscript has been judged scientifically suitable for publication and will be formally accepted for publication once it meets all outstanding technical requirements.

Within one week, you’ll receive an e-mail detailing the required amendments. When these have been addressed, you’ll receive a formal acceptance letter and your manuscript will be scheduled for publication.

An invoice for payment will follow shortly after the formal acceptance. To ensure an efficient process, please log into Editorial Manager at http://www.editorialmanager.com/pone/, click the 'Update My Information' link at the top of the page, and double check that your user information is up-to-date. If you have any billing related questions, please contact our Author Billing department directly at authorbilling@plos.org.

If your institution or institutions have a press office, please notify them about your upcoming paper to help maximize its impact. If they’ll be preparing press materials, please inform our press team as soon as possible -- no later than 48 hours after receiving the formal acceptance. Your manuscript will remain under strict press embargo until 2 pm Eastern Time on the date of publication. For more information, please contact onepress@plos.org.

Kind regards,

Corstiaan den Uil

Academic Editor

PLOS ONE

Additional Editor Comments (optional):

Reviewers' comments:

# Editor report: Acceptance letter

**DOI:** 10.1371/journal.pone.0241406.r004

**Authors:** Corstiaan den Uil

19 Oct 2020

PONE-D-20-23904R1 

Early indicators of intensive care unit bed requirement during the Covid-19 epidemic A retrospective study in Ile-de-France region, France 

Dear Dr. RIOU:

I'm pleased to inform you that your manuscript has been deemed suitable for publication in PLOS ONE. Congratulations! Your manuscript is now with our production department. 

If your institution or institutions have a press office, please let them know about your upcoming paper now to help maximize its impact. If they'll be preparing press materials, please inform our press team within the next 48 hours. Your manuscript will remain under strict press embargo until 2 pm Eastern Time on the date of publication. For more information please contact onepress@plos.org.

If we can help with anything else, please email us at plosone@plos.org. 

Thank you for submitting your work to PLOS ONE and supporting open access. 

Kind regards, 

PLOS ONE Editorial Office Staff

on behalf of

Dr. Corstiaan den Uil 

Academic Editor

PLOS ONE
